# LL-MAROCO: A Large Language Model-Assisted Robotic System for Oral and Craniomaxillofacial Osteotomy

**DOI:** 10.3390/bioengineering12060629

**Published:** 2025-06-09

**Authors:** Lai Jiang, Liangjing Shao, Jinyang Wu, Xiaofeng Xu, Xinrong Chen, Shilei Zhang

**Affiliations:** 1 Department of Oral and Cranio-Maxillofacial Surgery, Shanghai Ninth People’s Hospital, Shanghai Jiao Tong University School of Medicine, Shanghai 200011, China; jianglai@sjtu.edu.cn (L.J.); wujinyang7029@foxmail.com (J.W.); xuxiaofeng110@163.com (X.X.); 2 College of Stomatology, Shanghai Jiao Tong University, Shanghai 200011, China; 3 National Center for Stomatology, Shanghai 200011, China; 4 National Clinical Research Center for Oral Diseases, Shanghai 200011, China; 5 Shanghai Key Laboratory of Stomatology, Shanghai 200011, China; 6 Academy for Engineering and Technology, Fudan University, Shanghai 200433, China; ljshao22@m.fudan.edu.cn; 7 Shanghai Key Laboratory of Medical Image Computing and Computer Assisted Intervention, Shanghai 200030, China

**Keywords:** large language model, oral and craniomaxillofacial surgery, osteotomy, automatic surgery system, robot-assisted surgery system

## Abstract

Oral and craniomaxillofacial bone deformities necessitate treatment through osteotomy. Robot-assisted osteotomy appears promising in oral and craniomaxillofacial surgery, but it lacks sufficient intelligence and comprehensive integration of navigation tracking with surgical planning. This study aims to develop an intelligent surgical robot, based on the large language model ChatGPT-4, to enable autonomous planning for oral and craniomaxillofacial osteotomies. An autonomous surgical planning system driven by ChatGPT-4 was developed. Surgical plans were autonomously generated based on expert-defined prompts and surgical objectives. A deep learning framework was employed to match navigation-generated visual data with textual planning outputs. The generated plans were subsequently converted into executable instructions for robotic surgery. System precision, execution accuracy, and usability were experimentally validated through common osteotomies. An anonymous Likert scale assessed operational efficiency. The proposed system achieved a trajectory planning accuracy of 0.24 mm and an average robotic execution accuracy of 1.46 mm. The completion rates for two representative procedures, Le Fort I osteotomy and genioplasty, were 87% and 92%, respectively. Survey results confirmed process feasibility. The integration of a large language model with surgical robot advances intelligent, precise, and safe oral and craniomaxillofacial osteotomy procedures.

## 1. Introduction

Congenital, developmental, and acquired oral and craniomaxillofacial bone deformities can adversely affect patients’ esthetic appearance and functional capabilities, necessitating treatment through osteotomy performed [1]. To further reduce the incidence of postoperative complications and enhance patients’ quality of life, surgeons increasingly choose minimally invasive surgery (MIS) during procedures [2]. The craniomaxillofacial bones are small, irregularly shaped, and surrounded by important blood vessels and nerves, such as the brachial plexus and facial nerve, which navigate through the surrounding soft tissues in complex paths. Despite the availability of preoperative imaging, such as CT or MRI, as a reference, operations performed in the limited intraoperative field still demand considerable personal experience and advanced surgical skills from the surgeon.

The intelligent integration of surgical navigation systems with robotic platforms is regarded as a promising approach to address the mentioned technical challenges in the field of oral and craniomaxillofacial surgery [3,4]. However, the planning of robotic surgical procedures currently relies heavily on fundamental medical knowledge and clinical experience and is manually carried out by surgeons. This manual process often results in substantial repetitive work when planning similar surgical procedures, significantly impacting efficiency. Additionally, translating the planned surgical steps into robot-executable commands typically involves complex coordinate transformations and programming. This process often requires assistance from computer science specialists, which is difficult to obtain in clinical practice. Moreover, the preoperatively acquired imaging data has not been effectively integrated with navigation tracking and the high-precision pose adjustment of the robotic arm during surgery, which limits the flexibility of manipulation and prevents full utilization of the advantages of high degrees of freedom [5,6]. Therefore, there is an urgent need to optimize this process through the integration of intelligent algorithms and system-level solutions, thereby improving the efficiency and accuracy of surgical planning and robotic execution.

Regarding the autonomous generation of surgical sequence planning, existing solutions primarily focus on mathematical modeling, heuristic algorithms, and machine learning [7,8,9,10,11]. However, in clinical surgeries, the conditions of the surgical site and the specific needs of patients often change dynamically. On the one hand, acquiring sufficient training data for every potential surgical scenario would require a significant investment of time and effort. On the other hand, while specifically developed algorithmic models can provide tailored solutions for complex and diverse surgical tasks within certain medical specialties, these models must be continuously improved and updated to accommodate the evolution of clinical practices and changing patient needs. This process can result in substantial computational expenses [12].

Studies show that the development of intelligent surgical robots requires the incorporation of “commonsense” capabilities, including procedural knowledge, surgical commonsense, medical commonsense, and general commonsense [13]. Procedural knowledge, derived from surgical manuals, defines surgical stages and steps. Surgical commonsense, drawn from practical experience, facilitates the selection of operative techniques, adjustment of approaches, and understanding of causal relationships. Medical commonsense encompasses anatomical knowledge and the effects of surgical interventions on tissues, while general commonsense pertains to the use of tools and basic physical intuition. However, current systems face significant challenges in modeling these forms of tacit knowledge effectively.

Large language models (LLMs) inherently possess the requisite “commonsense” capabilities [14]. Through pretraining on extensive textual data and subsequent fine-tuning, LLMs acquire multimodal reasoning capabilities that extend beyond textual information, enabling them to infer and generate the required textual information based on given instructions [15]. Due to comprehensive training on large-scale textual datasets, LLMs require only minimal fine-tuning with domain-specific data to adapt to a wide range of task requirements [16]. Research indicates that, in addition to excelling in natural language understanding, generation, and reasoning tasks, LLMs are also effective in generating sequence planning text tailored to various scenarios by integrating multimodal information [17]. Using a function library created by controllers and natural language instructions, LLMs can infer and generate task sequences and content suitable for robotic execution [18]. ChatGPT-4 has been shown to successfully perform sequence planning for robotic assembly tasks by leveraging real-time captured visual image data and natural language prompts for guidance [19].

The application of LLMs in the field of surgical robotics remains in the exploratory stage. This study aims to develop a navigation-assisted surgical robotic system based on ChatGPT-4, integrated into a craniomaxillofacial robotic platform previously developed by the research team. The goal is to propose a novel, efficient, and highly adaptive intelligent system control strategy. In this context, a large language model-assisted robotic system, LL-MAROCO, was introduced for oral and craniomaxillofacial osteotomy. Leveraging the robust reasoning capabilities of ChatGPT-4, the system autonomously generates surgical sequence plans based on expert-defined prompts and surgical objectives. These text-based instructions are subsequently translated into executable programming code by downstream deep learning frameworks within the system. The ability of ChatGPT-4 to produce accurate and contextually appropriate instructions is closely tied to the quality and structure of the human-designed prompts. This study investigates effective prompting strategies to guide the generation of optimal textual outputs. Under the control of the instruction set generated by ChatGPT-4, osteotomy procedures were carried out using the craniomaxillofacial robotic system to validate the system’s precision and operational stability. In addition, a questionnaire survey was conducted to evaluate the LL-MAROCO system’s clinical feasibility, safety, and operational simplicity.

The contributions of this work are described as follows:

(1) To the best of our knowledge, LLMs are applied for the first time to generate autonomous surgical instructions for osteotomy planning. With appropriately designed prompts, clinically relevant surgical plans can be produced by the model.
(2) A control system integrating a surgical robotic platform with a navigation system is developed. Based on the generated instructions, the robotic system is able to perform task-specific actions aligned with pre-defined anatomical targets.
(3) Experiments conducted on a skull model demonstrate the feasibility of the proposed system for autonomous osteotomy. In addition, a questionnaire-based evaluation indicates satisfactory performance in terms of clinical reliability and operational effectiveness.

## 2. Materials and Methods

**Figure 1:**
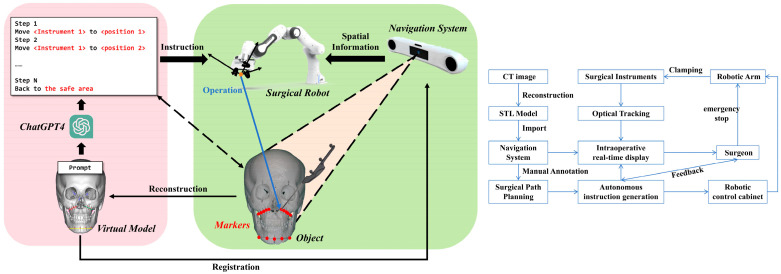
LL-MAROCO system configuration and control schematic diagram.

The framework of the proposed system, as shown in Figure 1, comprises a surgical robotic arm, an optical navigation system and the LLM ChatGPT-4. Cranial spiral CT scan data, acquired from clinical sources, is imported into the planning module in the navigation system, allowing specialized technicians to craft the osteotomy plan. Combining elaborate natural language prompts, the system generates an operational sequence for robot-assisted osteotomy. Once appropriate response texts are approved, they are translated into corresponding programming languages to generate command codes for operating the robotic arm. These commands are subsequently utilized to execute an osteotomy demonstration on the resin skull model. In this system, the surgical plan, model reconstruction, real-time imaging, and robotic operation are all closely integrated, with the key planning steps for the osteotomy task autonomously generated by ChatGPT-4, featuring an orderly upstream and downstream information exchange relationship. During this process, the surgeon can make an emergency stop to the system at any time to avoid safety hazards caused by malfunction or overload of the system.

### 2.1. Instruction Generation

**Figure 2:**
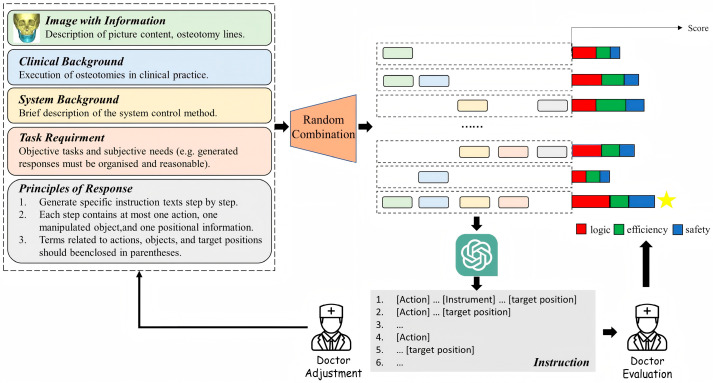
The pipeline for the generation of surgical instructions. Yellow star denotes the best combination of the instructions.

In this study, considering multiple factors such as the development quality and widespread application of existing LLMs, OpenAI’s ChatGPT-4 is selected as the representative for research. Throughout the study, patient privacy in terms of personal information will be strictly protected. The framework is integrated with a craniomaxillofacial surgical robotic system without requiring professional domain-specific fine-tuning of the existing ChatGPT-4, as shown in Figure 2.

Combining the characteristics of surgery with the application progress of LLMs in the medical field, surgical sequence operations can be simplified to the movements and positioning of specific surgical instruments, such as moving the reciprocating saw to the starting point of the marked osteotomy line or retracting it to a safe position. ChatGPT-4 generates text responses that include the actions of the robotic arm, the target objects, and their target positions based on natural language prompts and related task instructions. Orderly and reasonable replies are considered successful sub-task instructions for osteotomy. In other words, the instruction generation process can be conceptually understood as transforming high-level natural language prompts into a sequential list of robotic actions, thereby bridging human intentions and robotic execution. This modular approach ensures that each prompt corresponds to a parsable and executable robotic command. Since clinical demands often require customization, and the existing ChatGPT-4 cannot independently design the final position, the final movement of the bone piece to the target position is still manually set after the osteotomy.

In terms of designing prompt information, our initial prompts included CT reconstruction images, descriptions of osteotomy procedures, task requirements, and replying to principles. We set the following three principles for ChatGPT-4 responses:

1. Generate specific instruction texts step by step.
2. Each step contains at most one action, one manipulated object, and one positional information.
3. Terms related to actions, objects, and target positions should be enclosed in parentheses.

### 2.2. Multi-Space Registration

The system involves multiple coordinate systems, including the robotic arm base coordinate system O, the robotic arm end-effector coordinate system E, the patient image-guided coordinate system I, the reference frame coordinate system R, and the surgical tool coordinate system S. Among these, the transformation matrix between the robotic arm’s end-effector and its base is referred to as the robotic arm pose matrix. The surgical navigation subsystem utilizes preoperative CT imaging data of the patient to reconstruct a three-dimensional image, thereby establishing the image-guided space.

Spatial registration methods are used to determine the coordinate transformation between the reference frame coordinate system and the image-guided coordinate system. The spatial relationship between the surgical tool and the patient’s lesion area is then displayed in real time within the image-guided space. Guided by the surgical navigation subsystem, enabling the robot to assist in surgical operations requires managing multiple spatial coordinate transformations, i.e., achieving multi-space coordinate registration.

The goal of registration is to use the patient’s imaging data to guide the robotic arm in adjusting its target pose, determining the transformation matrix $T_{EO}$, which represents the mapping from coordinate system E to coordinate system O. This process involved aligning the patient’s space, image-guided space, and robotic workspace to enable precise intraoperative localization, ensuring accurate surgical tool positioning and high-quality procedure execution.

To enable real-time tracking of the robotic arm via the surgical navigation subsystem and ensure its operation aligns with the surgical planning results, the robotic arm’s target pose matrix $T_{EO}$ is a critical component of the system design. The key transformation matrices include the following:

$T_{IR}$: the transformation matrix from the image-guided coordinate system I to the reference frame coordinate system R;

$T_{RO}$: the transformation matrix from the reference frame coordinate system R to the robotic arm base coordinate system O;

$T_{IS}$: the transformation matrix from the image-guided coordinate system I to the surgical tool coordinate system S;

$T_{SE}$: the transformation matrix from the surgical tool coordinate system S to the robotic arm end-effector coordinate system E;

$T_{EO}$: the desired pose matrix for the robotic arm.

Each transformation matrix *T* consists of a rotation matrix and a translation matrix. To compute these transformation matrices, this study adopts a point-based least squares fitting method, specifically the singular value decomposition (SVD) technique.

The transformation relationship between the image-guided space and the robotic arm base is:

(1) $$T_{IO}=T_{RO}\cdot T_{IR}$$

On the other hand, the transformation relationship between the image-guided space and the end-effector is:

(2) $$T_{IO}=T_{EO}\cdot T_{SE}\cdot T_{IS}$$

From Equations (1) and (2), the desired pose of the robotic arm can be derived as:

(3) $$T_{EO}=T_{RO}\cdot T_{IR}\cdot T_{IS}^{-1}\cdot T_{SE}^{-1}$$

The reference frame and the robotic arm base are fixed, so $T_{RO}$ can be directly obtained. The spatial transformation matrix $T_{IR}$, which maps the image-guided space to the reference frame space, is determined by combining point registration and surface registration methods. $T_{SE}$ can be obtained by moving the surgical instrument and the robotic arm end-effector to the same fixed position and determining the coordinate transformation relationship between the instrument tip and the robotic arm’s end-effector center. $T_{IS}$ can be derived by tracking the reflective markers on the surgical instrument using an optical tracker. Therefore, based on the surgical planning results, the desired pose matrix of the robotic arm $T_{EO}$ can be calculated using Equation (3), as shown in Equation (4). Under the guidance of the surgical navigation system, the robotic arm can then be manipulated through inverse kinematics to ensure the surgical instrument follows the planned surgical path, thereby achieving high-quality surgical operations.

(4) $$T_{IR}=\left[\begin{array}{cc} \begin{array}{cc} 0.308371 & 0.951266\\ -0.948173 & 0.307338 \end{array} & \begin{array}{cc} 0.000355 & 0.835063\\ 0.080687 & 0.230211 \end{array}\\ \begin{array}{cc} 0.076645 & -0.025218\\ 0 & 0 \end{array} & \begin{array}{cc} 0.996739 & 0.245471\\ 0 & 1 \end{array} \end{array}\right]$$

### 2.3. Navigation System

**Figure 3:**
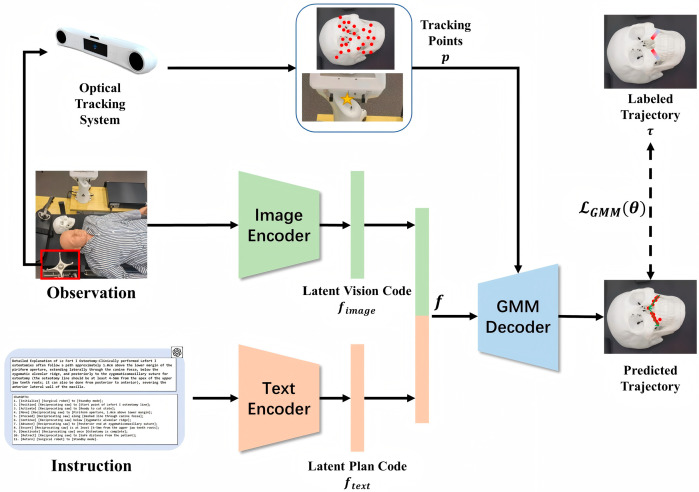
The pipeline of the deep learning framework to predict the trajectory.

A deep learning framework is designed to predict the trajectory points based on the information from the text instruction and the visual observation. The pipeline of the proposed framework is shown as Figure 3.

Given an observation I, the feature of the input image is extracted by ResNet-50 ($F_{Res50}$) as the image encoder. Meanwhile, the feature of the generated text instruction T is extracted by a transformer-based architecture ($F_{Trans}$). After that, the feature representation of the observation and instruction are fused by concatenating (⊕) the feature vectors, shown as Equation (5).

(5) $$f=F_{Res50}(I)⊕F_{Trans}(T)$$

Based on the tracking labels, the 3D points p on the target objects in the coordinate system of the optical tracking system are tracked. Furthermore, an MLP-based Gaussian mixture model (GMM) [20] is used to model the trajectory distribution based on the concatenated latent code f. The loss function of the GMM is the negative log-likelihood of the labeled trajectory obtained by surgeon τ, as Equations (6) and (7), where $\theta ={\left\{\alpha_{k},\mu_{k},\sigma_{k}\right\}}_{k=1}^{N}$ is the parameters of the GMM obtained by several MLPs (${MLP}_{k}$) from the input, $\sum_{k=1}^{N}{(\alpha }_{k}N(\mu_{k},\sigma_{k}))$ is the predicted distribution of the trajectory points and $N\left(\mu_{k},\sigma_{k}\right)$ is a Gaussian distribution.

(6) $$\theta_{k}=\left(\alpha_{k},\mu_{k},\sigma_{k}\right)={MLP}_{k}(f,p)$$

(7) $$L_{GMM}\left(\theta \right)=-E_{\tau }\log\sum_{k=1}^{N}\left(\alpha_{k}N(\mu_{k},\sigma_{k}\right))$$

Based on the predicted trajectory points in the coordination system of the optical tracking system $\tau^{OT}=\left\{x_{i},y_{i},z_{i}\right\}=\sum_{k=1}^{N}{(\alpha }_{k}N(\mu_{k},\sigma_{k}))$, the trajectory in the coordination system of the robot can be calculated as Equation (8), where the transformation of the coordination system $T_{OT}^{RB}$ is also obtained by the optical tracking system.

(8) $$\tau^{RB}=\tau^{OT}T_{OT}^{RB}$$

### 2.4. Experimental Platform

**Figure 4:**
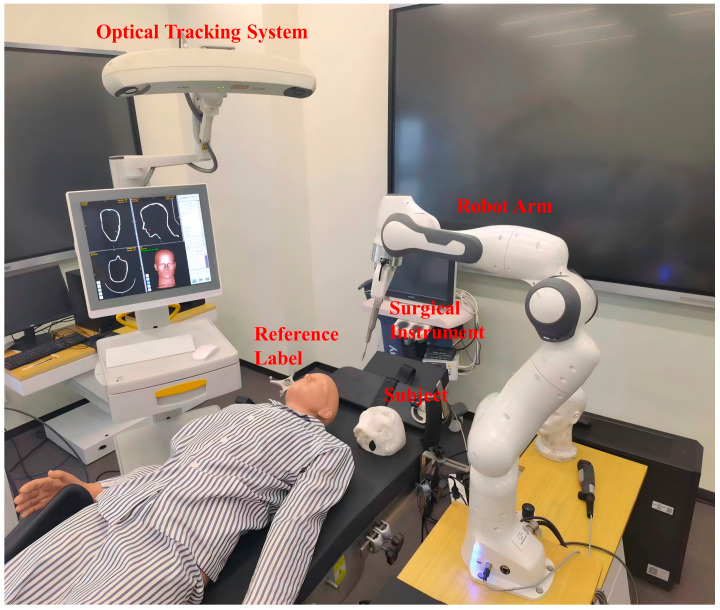
The implementation of the robot-assisted surgical system.

The robotic surgery system is implemented as Figure 4 shows. The NDI passive polaris spectra is utilized as the optical tracking system. The 7-DoF Franka Emika robot arm is used as the robot system. The code of the navigation module is implemented by Pytorch 1.13.1 and the GMM is trained on one NVIDIA RTX 4090 GPU (NVIDI, Santa Clara, CA, USA) for 500 epochs with Adam optimizer.

**Figure 5:**
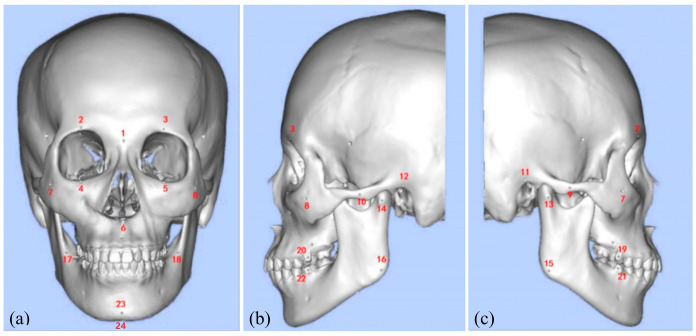
Virtual skull model with anatomical landmarks. (**a**) Frontal view; (**b**) left side view; (**c**) right side view. The anatomical landmarks corresponding to the numbered labels in the figure can be found in Table 1.

In this experiment, DICOM data from clinically acquired cranial spiral CT series were imported into CMF Robot Plan, where a three-dimensional cranial model was reconstructed and stored in STL format. The STL file of the virtual three-dimensional cranial model was imported into the design software Geomagic Studio 12.0 (Geomagic Inc., Morrisville, NC, USA). Referring to landmarks commonly used in craniofacial bone radiographic measurement and analysis, 24 anatomical landmarks for registration and accuracy testing were designed on the model as Table 1 and Figure 5 shows. These landmarks were displayed as cones with a diameter of 1 mm and a depth of 1 mm. The completed three-dimensional cranial model was imported into a 3D printer, Objet260 Connex3 (Stratasys Ltd., Eden Prairie, MN, USA), in STL format. It was processed using the 3D printing resin material MED620 (Stratasys Ltd., Eden Prairie, MN, USA) to create the experimental cranial model.

### 2.5. Evaluation of Surgery Performance

To evaluate the accuracy of the navigation system and the efficiency of the robot, operations are performed 10 times on the subject. The trajectory $\hat{\tau }=\{{\hat{x}}_{i},{\hat{y}}_{i},{\hat{z}}_{i}\}$ of the robot is recorded by the robot system with 15 fps. Meanwhile, the surgeon uses the surgical instrument with localization labels to operate on the subject and the trajectory $\tau =\left\{x_{i},y_{i},z_{i}\right\}$ is also obtained by the optical navigation system τ. The absolute position error $E_{o}$ of the trajectory points between $\hat{\tau }$ and $\tau^{RB}$, mentioned in Section 2.3, is utilized as Equation (9), to evaluate the stability of the robot control. Moreover, the accuracy of the trajectory planning $E_{p}$ is evaluated by the absolute position error of the trajectory points between $\hat{\tau }$ and τ as Equation (10).

(9) $$E_{o}=\left\|\hat{\tau }-\tau^{RB}\right\|$$

(10) $$E_{p}=\left\|\hat{\tau }-\tau \right\|$$

During robotic operations, the robot sometimes failed to complete the procedure because of spatial interference or extreme joint configurations. In addition, the control system includes an endpoint trajectory monitoring module that detected deviations exceeding 2 mm. When such deviations are identified, the system automatically triggered an error response and halted operation. The operator can also intervene at any time using an emergency stop button, providing redundant safety supervision. All instances of such errors are classified as unsuccessful path completions. Therefore, 20 experiments on each of 10 different situations for Le Fort I and genioplasty are performed and the success rate is recorded.

To assess the reliability and effectiveness of the LL-MAROCO system, a questionnaire survey is administered to 50 experienced craniomaxillofacial surgeons from the Department of Oral and Craniomaxillofacial Surgery, Ninth People’s Hospital, Shanghai Jiao Tong University School of Medicine (Shanghai, China). All 50 participating surgeons have over 5 years of clinical experience in oral and craniomaxillofacial surgery. The average age is 38.7 ± 6.4 years, with a range from 30 to 52 years. To reduce potential bias, participation in the questionnaire is anonymous, and no briefing is conducted regarding the expected outcomes. After viewing a video demonstration of the procedure, the surgeons rate each item on the evaluation form using a five-point Likert scale ranging from “strongly agree” to “strongly disagree”. The questions include the following:

1. The content of the reply made by ChatGPT-4 is easy to understand and logical.
2. The text generated by ChatGPT-4 for the decomposition of surgical robotic osteotomy tasks is safe and reasonable.
3. The process of the LL-MAROCO system as shown in the video is reasonable and convenient for you.
4. The actual osteotomy process of the robotic arm as shown in the video aligns with actual clinical practice.
5. If this type of control strategy can be promoted, you think it can protect patients’ rights and interests.
6. You are willing to use LL-MAROCO to perform robotic osteotomies yourself.

## 3. Results

### 3.1. Quality of Generated Instruction

The target procedure was exemplified by Lefort I osteotomy. Through repeated interaction with ChatGPT-4, we found that if a more comprehensive description of the clinical osteotomy procedure was provided, ChatGPT-4’s responses were less ideal. For example, during a Lefort I osteotomy, it erroneously identified a relatively irregular and extensive anatomical structure like the “zygomatic alveolar ridge” as a specific path.

To systematically investigate how the structure and content of prompt design affect the quality of instructions generated by ChatGPT-4, a controlled comparative experiment was conducted. The three following types of prompting strategies were tested:

Type A (minimal prompting): included only basic task commands and positional labels.

Type B (moderate prompting): included procedural context and anatomical image references in addition to basic commands.

Type C (detailed prompting): comprised a comprehensive clinical description of the surgical procedure.

Each prompt was used to generate a surgical plan for Le Fort I osteotomy. The generated responses were evaluated by two senior oral and craniomaxillofacial surgeons, each with more than seven years of clinical experience, from the Department of Oral and Craniomaxillofacial Surgery, Shanghai Ninth People’s Hospital, Shanghai Jiao Tong University School of Medicine (Shanghai, China), in order to partially eliminate subjective bias. The evaluation was based on the following criteria:

1. Instruction Accuracy (I-A): whether the generated instructions correctly matched the surgical paths and instruments.
2. Anatomical Appropriateness (A-A): whether relevant anatomical landmarks and target sites were correctly identified.
3. Step Coherence (S-C): whether a logical sequence was maintained between different procedural steps.
4. Terminological Precision (T-P): whether domain-specific terminology was used appropriately without semantic ambiguity.

**Figure 6:**
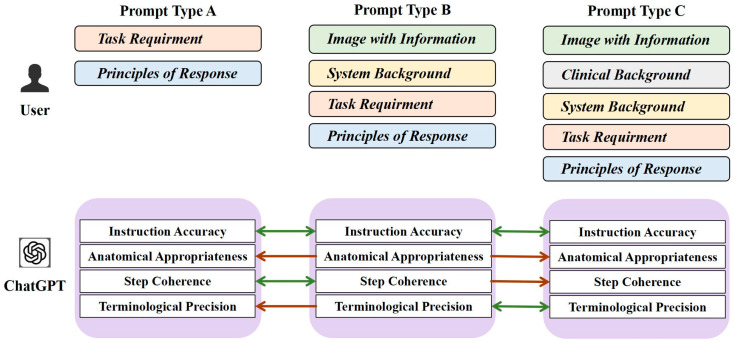
The systematic analysis of how varying prompts affect the quality of the generated instructions. Green two-way arrows indicate no significant difference, and red one-way arrows indicate a better effect than the object indicated.

**Figure 7:**
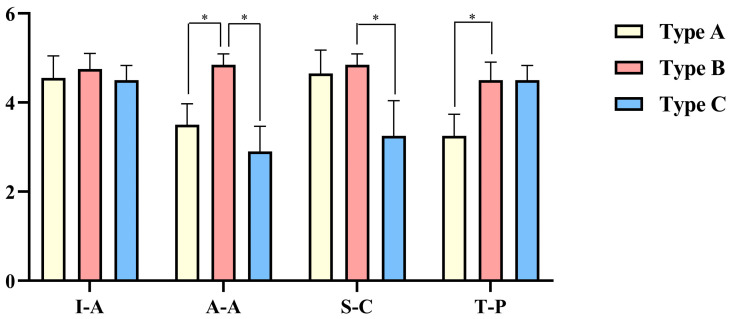
Average scores across different prompt types for each evaluation criterion. Asterisks indicate statistically significant differences (*p* < 0.05).

Each criterion was rated on a 5-point scale, and surgical plans were generated for ten distinct patients. Figure 6 illustrates the systematic analysis of how varying prompts affect the quality of the generated instructions. The scores from the two experts were denoted using “/” and were subjected to statistical analysis and comparison, as illustrated in Table 2 and Figure 7. In Figure 6, asterisks indicate statistically significant differences determined by Wilcoxon signed-rank test with Bonferroni correction (*p* < 0.05). Each bar in the chart represents the average score across the ten planning tasks. Type B prompts received the highest scores across all four evaluation metrics. Specifically, Type B prompts significantly outperformed both Types A and C in anatomical appropriateness, exceeded Type C in Step Coherence, and outperformed Type A in terminological precision.

Ultimately, the results of its interactive instruction generation were as follows:

User:

*I need to perform a maxillary Lefort I osteotomy in orthognathic surgery using a robot. In the control center we have marked the target position in the path: [A: Safe Position], [B: NL], [C: NR], [D: MoL], [E: MoR]. You are required to carry out safe and sensible robot sequence instruction planning.*

*(NL/NR: Left/Right Nasal lateral; MoL/MoR: Left/Right maxillary first Molar region)*

ChatGPT-4:

*1. **[Initialize]** the **[robot]**.*

*2. **[Move]** the **[osteotomy tool]** to **[A: Safe Position]**.*

*3. **[Move]** the **[osteotomy tool]** from **[A: Safe Position]** to **[B: NL]**.*

*4. **[Activate]** the **[osteotomy tool]** at **[B: NL]**.*

*5. **[Perform]** the osteotomy cut from **[B: NL]** to **[D: MoL]**.*

*6. **[Deactivate]** the **[osteotomy tool]** at **[D: MoL]**.*

*7. **[Move]** the **[osteotomy tool]** to **[C: NR]**.*

*8. **[Activate]** the **[osteotomy tool]** at **[C: NR]**.*

*9. **[Perform]** the subsequent osteotomy cut from **[C: NR]** to **[E: MoR]**.*

*10. **[Deactivate]** the **[osteotomy tool]** at **[E: MoR]**.*

*11. **[Move]** the **[osteotomy tool]** from **[E: MoR]** to **[A: Safe Position]**.*

**Figure 8:**
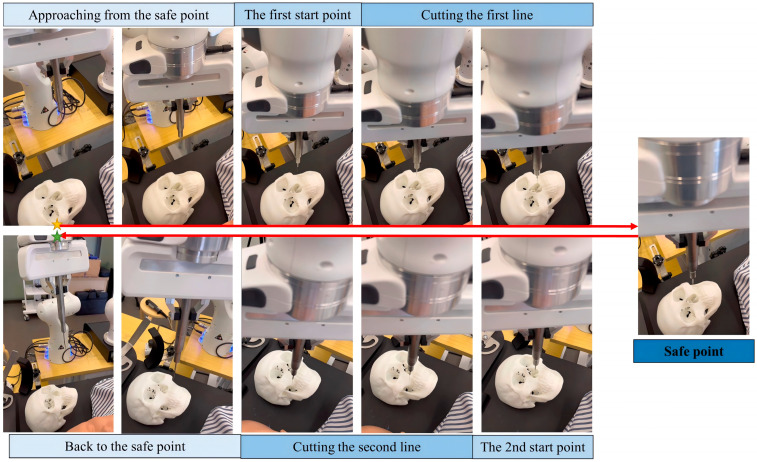
Time-lapse sequence showing the autonomous Le Fort I osteotomy: the instrument is initialized, the bilateral osteotomy is executed along the planned trajectory, and the tool is finally retracted to the safe position. The yellow star denotes the start of the process, while the green one denotes the end frame.

The robotic arm execution flow corresponding to the autonomously generated text instructions is shown in Figure 8.

### 3.2. Quantitative Results: Trajectory Accuracy and Procedural Completion

In 10 repeated trials, the mean absolute error between the navigation-generated path and the surgeon-defined reference trajectory was 1.46 mm. After multi-space registration, the robot end-effector followed the same path with a mean control error of 0.24 mm, as shown in Table 3. For the two representative procedures, Le Fort I osteotomy and genioplasty, twenty demonstrations were conducted in each of ten anatomical scenarios, yielding a total of four hundred runs. Le Fort I osteotomy recorded 174 successful executions out of 200, giving a completion rate of 87 per cent, whereas genioplasty achieved 184 successes out of 200, corresponding to 92 per cent, as detailed in Table 4. These results indicate that LL-MAROCO can perform autonomous osteotomies with high positional accuracy and maintain reliable task completion across different surgical requirements.

The proposed method was compared with three methods in terms of the average success rate and average trajectory deviation, as measured against expert-determined standards, in robotic surgeries for Le Fort I osteotomy and genioplasty. Three compared methods included BC-RNN [21], BC-GPT [22] and BC-R3M [23], which were, respectively, goal-instructed variants of Behavior Clone [24] using RNN, GPT-based transformer architectures and R3M visual representations. The proposed method achieved state-of-the-art performance based on the quantitative results summarized in Table 5.

### 3.3. Qualitative Results: Questionnaire-Based Subjective Feedback from Surgeons

**Figure 9:**
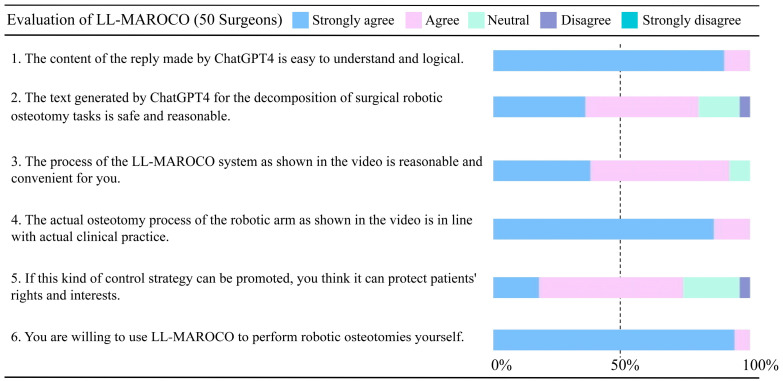
Statistics on the results of questionnaires.

The survey results indicate that the feedback was predominantly positive, with detailed results presented in Table 6 and Figure 9.

To quantitatively assess the consistency of subjective responses, the standard deviation (SD) of each Likert item was calculated. The SD values for Q1–Q6 were as follows: 0.27, 0.79, 0.72, 0.34, 0.83, and 0.23, respectively. These results suggested that most participants gave highly convergent responses, particularly regarding the system’s clarity (Q1), procedural conformity (Q4), and willingness to use (Q6). All 50 experienced oral and craniomaxillofacial surgeons surveyed strongly agreed/agreed that the content generated by ChatGPT-4 was easy to understand and logical (Q1), and that the LL-MAROCO system’s procedural flow was essentially consistent with the standard procedures of craniomaxillofacial surgical osteotomy (Q4). This is crucial for further application research and clinical trials. LL-MAROCO shows no significant deviations from current practice standards, allowing even surgeons without extensive computer technology knowledge to easily understand and rigorously follow the autonomously generated steps. Additionally, 92% of surgeons found the use of LL-MAROCO convenient (Q3), and 100% of surveyed surgeons expressed a strong willingness to use it (Q6). This suggests excellent prospects for the system’s efficiency in use and widespread adoption. Two surgeons raised concerns regarding system safety (Q2) and protection of patients’ rights (Q6).

## 4. Discussion

### 4.1. Research Synthesis and LL-MAROCO’s Performance

Digital technologies have gradually been developed and integrated into oral and craniomaxillofacial surgery over the past 40 years, such as 3D-printed personalized guides [25,26], navigation system [27,28], virtual reality (VR), and augmented reality (AR) [29,30]. However, human-performed surgical procedures still face unavoidable limitations. The robotic-assisted surgical system (RASS) offers unique advantages, including providing motion angles and stability that cannot be achieved by human hands, integrating imaging and visualization technologies, synthesizing multi-source intraoperative information to enhance real-time feedback, performing repetitive tasks, and enabling remote surgery [3]. These systems have already been widely applied in specialties such as neurosurgery and urology [31].

Based on these developments, a workflow integrating surgical navigation with robotic execution has been proposed to overcome the constraints associated with manual operations. The current workflow can be summarized as follows. First, preoperative image data, such as CT scans, is utilized in the surgical navigation system to perform surgical planning. Second, spatial registration is achieved among the patient coordinate system, the image-guidance coordinate system, and the robotic workspace using the spatial positioner of the surgical navigation system. Third, the surgical steps derived from the surgical plan are translated into a sequence of executable commands interpretable by the robotic system. Finally, the surgical robot performs the operation according to these commands, with the navigation system providing real-time tracking to ensure the safety and precision of the procedure.

Although this integrated workflow offers a technical pathway toward intelligent surgery, its clinical application in oral and craniomaxillofacial procedures remains at an early stage. A significant gap persists between theoretical feasibility and clinical translation. To date, experiments performed on resin models and porcine skulls have demonstrated the feasibility of robotic autonomous drilling [32,33], as well as the movement and positioning of bone segments in bone tissues [34,35]. Additionally, a collaborative human–robot control method, based on force feedback and optical navigation, has been shown to be effective for completing predefined osteotomy paths in Lefort I osteotomy and genioplasty [36,37], combining the surgeon’s subjective judgment with the high precision and stability of robotic arm outputs.

In recent years, studies on robotic systems, similar to the present research, have been progressively conducted across various domains, yielding promising outcomes. Naoki Wake et al. proposed a task planning method based on customized prompting using ChatGPT, which enables efficient translation of natural language instructions into multi-step robotic actions without the need for prior operational experience, significantly enhancing human–robot interaction in few-shot environments [38]. Yang Ye et al. integrated ChatGPT into robotic control systems to facilitate natural language-based communication, thereby improving human trust in collaborative tasks [39]. Yubing Gao et al. investigated the integration of ChatGPT with intelligent vehicle systems, demonstrating enhanced interaction experience and supporting flexible, real-time decision-making [40]. Analogously, the present study leverages the robust reasoning capabilities of ChatGPT-4 in conjunction with optical navigation-based control strategies to facilitate enhanced information transmission. This integration supports autonomous sequence planning and accurate execution of craniomaxillofacial osteotomies, constituting a novel application in the field of oral and craniomaxillofacial surgery.

In terms of robotic surgical precision, according to Bell et al. at the Royal Infirmary of Glasgow, United Kingdom, clinical findings from 62 cases of robot-assisted unicompartmental knee arthroplasty demonstrated that an osteotomy accuracy of 1.0–1.5 mm is required to ensure satisfactory surgical outcomes [41]. Moreover, the majority of current literature on bone-cutting surgical robots suggests that an error tolerance within 2 mm is generally accepted as the clinical threshold [42,43,44]. Experimental results from this study indicated that the LL-MAROCO system meets these accuracy standards when performing craniomaxillofacial osteotomies and achieves a high task completion rate. Compared to state-of-the-art methods such as BC-RNN, BC-GPT, and BC-R3M, the LL-MAROCO system demonstrated superior performance in both procedural success rates and trajectory accuracy during Le Fort I osteotomy and genioplasty. This advantage arises from the synergistic integration of high-level visual feature representation and robust natural language-driven action planning, leveraging the inherent generalization and reasoning capabilities of large language models to generate context-aware instructions. These capabilities enable more adaptive and targeted execution of complex osteotomy tasks.

In addition, its usability and clinical relevance were unanimously endorsed by experienced surgical professionals, drawing considerable interest. This is of substantial importance for future system deployment, as it could facilitate smoother learning curves and enhance operational efficiency during training and practical implementation. Although the proposed system demonstrates promising capabilities in simulated clinical scenarios, complete elimination of surgeon involvement remains unfeasible at this stage. Critical decisions such as defining final surgical objectives and protecting anatomical structures outside the lesion area must still rely on the expertise of qualified surgeons. Furthermore, ensuring a clear operative field, particularly in the presence of bleeding or soft tissue interference, and maintaining continuous surgeon supervision are key challenges. These issues require targeted solutions in future studies involving live animal models or real clinical scenarios.

### 4.2. Ethical Considerations

The integration of LLMs into surgical robotic planning systems raises important ethical considerations. First, the use of AI-driven systems in surgery introduces the potential for bias, particularly in complex or ambiguous clinical scenarios. To mitigate this risk, the system should be deployed only in a closed-loop configuration, where real-time human oversight and robotic navigation feedback remain essential safeguards. Second, all simulated surgical plans in this study were anonymized. Nonetheless, future clinical deployment would require strict compliance with data protection regulations and informed consent protocols. Finally, the ethical responsibility in human–robot collaborative surgery must remain clearly defined. Although the LL-MAROCO system was designed to generate surgical instructions in a structured and supervised manner, the underlying language model lacks medical licensure and domain-specific accountability. Therefore, all outputs must be regarded as advisory and require validation by qualified medical professionals prior to any clinical application. At the current stage, the system serves as an intelligent assistant under full surgical supervision. Surgical accountability and final decision-making remain the responsibility of the attending surgeon.

## 5. Conclusions

This study proposes a framework integrating LLM ChatGPT-4 into robotic systems to assist in the autonomous planning and execution of osteotomy tasks in orthognathic surgery. By designing structured prompts and incorporating robotic hardware with navigation platform control systems, the feasibility of generating surgical instructions via ChatGPT-4 was validated. Experiments conducted on skull models demonstrated the system’s capability to autonomously complete basic osteotomy tasks. Moreover, expert evaluation questionnaires indicated preliminary practical value in terms of clinical reliability, usability, and operational efficiency. The advancement in autonomy is expected to significantly reduce reliance on surgeons’ specialized knowledge, decrease time costs during treatment, alleviate surgeons’ workload, and promote higher levels of robotic intelligence in oral and craniomaxillofacial surgical procedures.

Despite these advancements, the current system has certain limitations. First, regarding instruction generation under different prompting strategies, although the existing ChatGPT-4 performs well in processing multimodal information, it lacks sufficient comprehension when dealing with highly specialized images and texts. More intelligent systems require further adaptation and fine-tuning of LLMs. Additionally, the current system does not yet accommodate intraoperative soft tissue dynamics, exhibiting limited adaptability to unstructured changes during procedures. Furthermore, the planning of fully personalized surgical plans still depends on the judgment and expertise of professional surgeons, and comprehensive replacement by current LLMs and robotic systems remains unattainable.

Future research will focus on the integration of multimodal information, including imaging data, intraoperative endoscopic images, and biomechanical simulations, to enhance the system’s contextual understanding and adaptability. Subsequent work will also aim to expand the scope of surgical tasks, strengthen real-time response capabilities, and validate performance in more complex and diverse clinical scenarios.

## Figures and Tables

**Table 1:** The anatomical landmarks.

Number	The Anatomical Landmark	Number	The Anatomical Landmark
1	Nasion	13	Right Lateral Condyle Point
2	Right Supraorbital Foramen	14	Left Lateral Condyle Point
3	Left Supraorbital Foramen	15	Right Gonion
4	Right Infraorbital Margin	16	Left Gonion
5	Left Infraorbital Margin	17	Right Mandibular Ramus Bend
6	Anterior Nasal Spine	18	Left Mandibular Ramus Bend
7	Right Zygion	19	Maxillary right first molar buccal point (FDI 16)
8	Left Zygion	20	Maxillary left first molar buccal point (FDI 26)
9	Right Zygomatic Arch Prominence	21	Mandibular right first molar buccal point (FDI 46)
10	Left Zygomatic Arch Prominence	22	Mandibular left first molar buccal point (FDI 36)
11	Right Auricular Point	23	Pogonion
12	Left Auricular Point	24	Menton

**Table 2:** Scoring results from two expert evaluators on instruction generation quality for each prompt type.

	Type A										Type B										Type C									
I-A	5/5	4/5	5/5	4/5	4/4	3/4	4/5	5/5	4/5	5/5	4/5	5/5	5/5	5/5	5/5	4/5	5/5	4/5	4/4	5/5	4/4	4/5	5/4	5/4	5/4	5/5	5/4	5/4	4/4	5/5
A-A	3/3	3/4	4/3	3/3	3/4	4/5	4/4	3/4	3/4	3/3	5/5	4/5	5/5	5/4	5/4	5/5	5/5	5/5	5/5	5/5	3/3	3/3	3/3	3/2	3/3	2/3	4/3	4/4	2/2	2/3
S-C	3/4	4/5	3/5	5/5	5/5	5/5	5/5	4/5	5/5	4/5	5/5	5/5	4/5	5/5	5/5	5/5	5/5	5/5	4/5	4/5	4/3	4/4	4/4	4/3	5/4	3/3	2/3	2/2	3/2	3/3
T-P	3/3	2/3	3/3	4/4	3/3	3/4	4/4	3/3	3/4	3/3	4/5	4/4	5/5	4/5	4/4	5/5	5/4	5/4	5/5	4/4	4/5	4/4	5/4	5/5	4/4	4/5	4/5	5/5	5/4	5/4

**Table 3:** The absolute position errors of the trajectory points.

Metrics	1	2	3	4	5	6	7	8	9	10	Avg.
$E_{o}$/mm	0.23	0.34	0.18	0.25	0.21	0.23	0.22	0.31	0.19	0.23	0.24
$E_{p}$/mm	1.45	1.98	1.23	1.32	1.38	1.41	1.43	1.67	1.32	1.38	1.46

**Table 4:** The completion rate of the operation process.

Target	1	2	3	4	5	6	7	8	9	10	Avg.
Le Fort I	85%	90%	80%	95%	85%	85%	90%	85%	95%	80%	87%
Genioplasty	90%	95%	90%	90%	95%	95%	90%	90%	90%	95%	92%

**Table 5:** Comparative success rates and trajectory deviations of the proposed method and state-of-the-art methods for Le Fort I osteotomy and genioplasty. The best results are bolded.

Methods	Success Rate		$E_{p}$/mm
	Le Fort I	Genioplasty	
BC-RNN [21]	71%	76%	1.68
BC-GPT [22]	82%	85%	1.52
BC-R3M [23]	80%	83%	1.61
Ours	**87%**	**92%**	**1.46**

**Table 6:** The questionnaire results.

Options	Q1	Q2	Q3	Q4	Q5	Q6
Strongly agree	45	18	19	43	9	47
Agree	5	22	27	7	28	3
Neutral	0	8	4	0	11	0
Disagree	0	2	0	0	2	0
Strongly disagree	0	0	0	0	0	0

## Data Availability

The original contributions presented in this study are included in the article. Further inquiries can be directed to the corresponding authors.

## Abbreviations

The following abbreviations are used in this manuscript:

AR	Augmented Reality
CAD/CAM	Computer-Aided Design/Computer-Aided Manufacturing
CT	Computed Tomography
DICOM	Digital Imaging and Communications in Medicine
FDI	Fédération Dentaire Internationale (ISO Tooth Numbering System)
GMM	Gaussian Mixture Model
LLM	Large Language Model
MIS	Minimally Invasive Surgery
MLP	Multi-Layer Perceptron
MRI	Magnetic Resonance Imaging
RASS	Robotic-Assisted Surgery System
STL	Standard Tessellation Language (File Format)
SVD	Singular Value Decomposition
VR	Virtual Reality
